# Does Malaria Affect Placental Development? Evidence from *In Vitro* Models

**DOI:** 10.1371/journal.pone.0055269

**Published:** 2013-01-31

**Authors:** Alexandra J. Umbers, Danielle I. Stanisic, Maria Ome, Regina Wangnapi, Sarah Hanieh, Holger W. Unger, Leanne J. Robinson, Elvin Lufele, Francesca Baiwog, Peter M. Siba, Christopher L. King, James G. Beeson, Ivo Mueller, John D. Aplin, Jocelyn D. Glazier, Stephen J. Rogerson

**Affiliations:** 1 Department of Medicine, University of Melbourne, Melbourne, Victoria, Australia; 2 Vector Borne Disease Unit, Papua New Guinea Institute of Medical Research, Goroka, Southern Highlands Province, Papua New Guinea; 3 Institute for Glycomics, Griffith University, Southport, Queensland, Australia; 4 Infection and Immunity Division, Walter and Eliza Hall Institute, Parkville, Victoria, Australia; 5 Center for Global Health, Case Western Reserve University, Cleveland, Ohio, United States of America; 6 Center for Immunology, Macfarlane Burnet Institute of Medical Research and Public Health, Melbourne, Victoria, Australia; 7 Barcelona Center for International Health, University of Barcelona, Barcelona, Spain; 8 Maternal and Fetal Health Research Centre, Institute of Human Development, University of Manchester, Manchester, United Kingdom; 9 St. Mary's Hospital, Central Manchester University Hospitals NHS Foundation Trust, Manchester Academic Health Science Centre, Manchester, United Kingdom; University of Copenhagen, Denmark

## Abstract

**Background:**

Malaria in early pregnancy is difficult to study but has recently been associated with fetal growth restriction (FGR). The pathogenic mechanisms underlying malarial FGR are poorly characterized, but may include impaired placental development. We used *in vitro* methods that model migration and invasion of placental trophoblast into the uterine wall to investigate whether soluble factors released into maternal blood in malaria infection might impair placental development. Because trophoblast invasion is enhanced by a number of hormones and chemokines, and is inhibited by pro-inflammatory cytokines, many of which are dysregulated in malaria in pregnancy, we further compared concentrations of these factors in blood between malaria-infected and uninfected pregnancies.

**Methodology/Principal Findings:**

We measured trophoblast invasion, migration and viability in response to treatment with serum or plasma from two independent cohorts of Papua New Guinean women infected with *Plasmodium falciparum* or *Plasmodium vivax* in early pregnancy. Compared to uninfected women, serum and plasma from women with *P. falciparum* reduced trophoblast invasion (P = .06) and migration (P = .004). *P. vivax* infection did not alter trophoblast migration (P = .64). The *P. falciparum*-specific negative effect on placental development was independent of trophoblast viability, but associated with high-density infections. Serum from *P. falciparum* infected women tended to have lower levels of trophoblast invasion promoting hormones and factors and higher levels of invasion-inhibitory inflammatory factors.

**Conclusion/Significance:**

We demonstrate that *in vitro* models of placental development can be adapted to indirectly study the impact of malaria in early pregnancy. These infections could result in impaired trophoblast invasion with reduced transformation of maternal spiral arteries due to maternal hormonal and inflammatory disturbances, which may contribute to FGR by limiting the delivery of maternal blood to the placenta. Future prevention strategies for malaria in pregnancy should include protection in the first half of pregnancy.

## Introduction

In endemic areas, *Plasmodium falciparum* malaria during pregnancy is the leading preventable cause of low birth weight (LBW) and neonatal mortality, largely due to fetal growth restriction (FGR) [1], [2]. The pathogenic mechanisms underlying malaria-associated FGR are poorly characterized, but may include placental insufficiency due to poor placental development [3]. Peak prevalence of maternal *P. falciparum* infections occurs between 13 and 18 weeks’ gestation [3]. This period coincides with the establishment of the placental circulation, when a sub set of placental cells, extravillous trophoblasts (EVT) invade and migrate through the decidua and transform maternal spiral arteries to increase placental blood supply [4], [5]. EVT invasion refers specifically to the invasion of placental cells into the maternal decidua and spiral arteries, while migration refers to movement of EVTs through the decidual extra cellular matrix [6]. The regulation of trophoblast invasion involves a complex network of cytokines, chemokines, hormones, and cellular interactions between invading placental tissue and maternal immune cells resident in the decidua and the vascular endothelium of spiral arteries [7], [8], [9], [10]. Adequate trophoblast invasion is essential for the establishment of appropriate placental function and successful fetal growth. Impaired trophoblast invasion is associated with placental insufficiency and FGR [11], especially in preeclampsia, a hypertensive disorder that shares important pathophysiological features with malaria in pregnancy [3].

Epidemiological and ultrasound studies have demonstrated that *P. falciparum* malaria infection in the first half of gestation increased the risk of LBW or other parameters of restricted fetal growth [12], [13], [14], [15], [16], [17], suggesting infection in early pregnancy negatively affects fetal growth. Ultrasound studies have shown impaired utero-placental blood flow, a hallmark of impaired early placental development, and this predicted LBW or episodes of fetal growth restriction in pregnant women with *P. falciparum* infection [15], [18].

The pathogenic basis of LBW due to maternal *P. vivax* infection [19] is even less well understood than *P. falciparum*. While there is some indirect evidence for placental binding of *P. vivax* [20], [21] the only *ex vivo* study to date suggests pathological mechanisms independent of placental malaria [22]. Understanding the impact of *P. vivax* on early placental development may reveal pathogenic mechanisms of FGR common to both parasite species.

Taken together these clinical observations support the theory that infection with *P. falciparum,* and perhaps *P. vivax* in early pregnancy, might impair placental development. The pathological mechanisms underlying these observations are unknown, but we hypothesize that shallow EVT invasion or migration into the maternal decidua and spiral arteries could explain the link between early malaria infection, poor placental development and FGR.

Placental development in laboratory animal models differs considerably from that of human pregnancy [23], and although rodent models may provide clues about malaria during human pregnancy [24], their utility as models of human placental development remains controversial.

The development of intrinsically invasive or migratory first trimester EVT cell lines circumvents some of the limitations associated with the use of animal models for early pregnancy studies and importantly, the ethical and practical considerations associated with the use of primary pregnancy tissues from women. EVT cell lines have been applied to *in vitro* invasion assays to identify factors that regulate placental development, the process of invasion and the effects of various factors in maternal blood from complicated pregnancies [25], [26]. In this study, we aimed to determine if this system could be utilized to indirectly assess the potential effects of malaria in early pregnancy.

The potential impact of *P. falciparum* and *P. vivax* malaria infection on placental development was examined by testing serum or plasma from two cohorts of Papua New Guinean (PNG) women with and without malaria infection in peripheral blood at their first antenatal presentation for the ability to impair invasion, migration and viability of a first trimester EVT cell line. We further compared maternal serum concentrations of hormones, cytokines and chemokines which are dysregulated in malaria in pregnancy [27], [28] and are known to modulate trophoblast invasion [11], between *P. falciparum*-infected and uninfected women.

## Methods

### Study Design

Cohort 1: Between 2005 and 2007, pregnant women at first antenatal visit to Alexishafen Health Centre, Madang, PNG were enrolled following written informed consent. Peripheral blood was collected (BD vacutainer, UK), hemoglobin measured by Hemocue (Hemocue, Quest Diagnostics, Madison, USA), and serum was separated and stored at −80°C. Gestational age was determined by reported last menstrual period and fundal height. Cohort 2: Pregnant women in the Madang area were enrolled into a malaria prevention trial between 2009 and 2011, following written informed consent. Peripheral blood was collected into Lithium Heparin tubes (BD) and plasma was separated and stored at −80°C. Gestational age was determined by last menstrual period and fundal height, and confirmed by ultrasound scan. The Medical Research Advisory Council of PNG and the Human Research Ethics Committee, Melbourne Health, Australia approved both studies. In both cohorts, placental malaria, birth weight and gestational age at delivery were measured as described previously [29].

### Diagnosis of Malaria Infection

Peripheral parasitemia and species identification were determined by microscopy of giemsa-stained blood smears by at least two qualified microscopists. Thick films were examined for 200 high-powered fields before being declared infection-negative. Slides were scored as light-microscopy-positive for an individual *Plasmodium* species, if the species was detected independently by at least two microscopists. Parasite densities were recorded as the number of parasites per 200–500 white blood cells and converted to the number of parasites/microliter assuming 8,000 white blood cells/microliter (Genton et al; 1995). Final parasite densities were obtained by calculating the geometric mean of positive reads.

### Inclusion Criteria

In the first cohort, we selected samples from women recruited between 16 and 22 weeks’ gestation with confirmed *P. falciparum* infections, regardless of parasitemia. Women were gravidity-matched with equal numbers of malaria-uninfected women as controls. In keeping with other studies [25], [30], pooled sera were tested to control for high inter-donor variability and varied gestational ages. After pooling, sera were aliquoted into appropriate volumes and stored frozen for individual experiments.

To address the effect of individual variability on trophoblast migration, we selected an additional cohort including women with an ultrasound confirmed gestational age of 20 or fewer weeks. Women with microscopically confirmed moderate to high asexual blood stage infection with *P. falciparum* (0.1–5.0% parasitemia) or *P. vivax* (any parasitemia) were matched by gestational age and gravidity with uninfected women. Twenty one percent of *P. falciparum* infections were scanty (≤.01% parasitemia) and therefore were excluded.

### Cell Line and Culture

The HTR8/SVneo EVT cell line was used to measure the effect of pooled early pregnancy serum on trophoblast invasion. The HTR8/SVneo line was derived from primary first trimester explant cultures and immortalized by transfection with the SV40 virus [31]. HTR8/SVneo express characteristic EVT markers and cytokine receptors and exhibit intrinsic invasion properties through 3D Matrigel™ in response to chemo-attractant factors in serum. Cells were cultured as previously described [31].

To measure the effect of plasma on trophoblast migration, the Swan 71 EVT cell line was used. EVT were isolated from a normal first trimester placenta and transformed with human telomerase reverse transcriptase [32]. Swan 71 cells display typical characteristics of primary EVT including CK7 and HLA-G expression, hCG secretion and expression of cytokines and growth factors, including IL-1β, IL-6, IL-8, similar to the profile of the HTR8/SVNeo line [32].

### Invasion Assay

Trophoblast invasion was measured using a commercial invasion assay kit (Chemicon, Australia). Confluent HTR8/SVneo were treated with trypsin and seeded in triplicate on Matrigel™ coated porous polycarbonate membrane inserts in serum reduced medium (SRM: RPMI 1640 supplemented with 1% penicillin/streptomycin solution, 200 mM L-glutamine and 1% fetal bovine serum (FBS), all supplied by Gibco); medium alone; medium supplemented with 10% vol of pooled serum from infected or uninfected women, or medium with 10 ng/ml TGFβ (positive control; from Peprotech, USA, gifted from R. Luwor). The lower chamber contained the respective treatments in complete medium (supplemented RPMI 1640 with 5% FBS). After 48 h incubation at 37°C, invasive cells that had adhered to the lower chamber were detached, lysed, and quantified according to the manufacturer’s instructions. The invasion assay using pooled serum was repeated three times, in triplicate.

### Migration Assay

Trophoblast migration in response to treatment with individual plasma samples was measured using a scratch assay. Swan 71 cells were cultured to 90% confluence as previously described [32], trypsinised, washed and seeded into 6 well dishes in serum free medium (SFM, 50∶50 DMEM/Ham’s F12 medium and 1% penicillin/streptomycin/L-glutamine) supplemented with 10% FBS (Invitrogen) overnight. At 90% confluence (0 h), a cross was scratched into each well. Wells were treated in duplicate with control treatments (10% FBS as migration promoter, and 500 ng/ml bacterial lipopolysaccharide (LPS, Sigma) as migration inhibitor [33]), with 10% plasma from individuals in SFM, or were untreated (SFM alone). To avoid potential bias, the operator was blinded to the malaria infection status of plasma. The scratched area was imaged using the Olympus IMX70 inverted microscope and a 4x objective and tiling software (Image Pro-insight, Media Cybernetics, Bethesda, MD). Plasma treatments were randomly allocated to one of four independent experiments, each with control and untreated conditions. For each treatment, migration into the scratch was measured at three locations. Fixed size tiled images taken at the same location at 0, 6 and 24 h for three independent scratch points were uploaded to ibidi website (www.ibidi.com). The percent of cell coverage (versus the scratched area) in each frame was determined using the WIMscratch image analysis platform (WIMASIS GmbH, Munich, Germany). The percent of cell coverage in each frame at 0 h was subtracted from subsequent time points at respective locations to give the percentage increase in coverage, as a proxy for migration, over time. The mean of the three migration replicates of each treatment was used for analysis.

### Alamar Blue Viability Assay

The vital dye alamarBlue***®*** (Invitrogen; AB) was used to establish trophoblast cell viability and test for changes of metabolic activity as expressed by cytoplasmic reducing power. This fluorescence or colorimetric assay is based on cellular uptake and metabolism of AB, where the cytotoxicity of a treatment relative to that of the control is indicated by the fractions of reduced and oxidized dye [34]. The percentage of AB reduced was calculated according to the manufacturer’s instructions. The assay was highly reproducible (mean ± standard deviation [SD] intra-assay variation of 8±7%).

To test the effect of pooled serum from *P. falciparum* -infected and uninfected women (cohort 1), HTR8/SVneo cells were seeded in triplicate in SRM. Following adherence, cells were incubated with either complete medium, complete medium supplemented with 10% *P. falciparum-* infected or uninfected pooled serum, or in SRM at 37°C in 95% air, 5% CO_2_ for 48 h. To determine the effect of 48 h treatment on trophoblast viability, washed cells were incubated with fresh complete medium with 10% vol AB for 4 h, and absorbance measured. Trophoblast activity was expressed as relative fold change in AB reduction, normalized to the complete media control treatment. The assay using pooled serum was repeated three times, in triplicate.

A modified version of the same assay was used to test the effect on Swan 71 cells of plasma collected from individuals with or without *P. falciparum* infection (cohort 2). Cells were seeded in triplicate and incubated with complete medium, or SFM supplemented with 10% plasma from uninfected or *P. falciparum-*infected women for 24 h at 37°C in 95% air, 5% CO_2_. Following treatment, medium was spiked with 10% AB and incubated for a further 4 h, and fluorescence measured according to manufacturer’s instructions. The assay was performed for multiple plasma samples in triplicate. Two uninfected plasma samples were excluded due to insufficient volumes.

### ELISA and Multiplex Cytokine Arrays

To compare concentrations of invasion-stimulatory and -inhibitory hormonal and cytokine modulators in pooled serum from malaria-infected and uninfected women in early pregnancy, insulin-like growth factor-I (IGF-I), IGF-II and human chorionic gonadotrophin (hCG) were measured once by ELISA (DSL, Texas and DRG, New Jersey, USA) in triplicate according to the manufacturer’s instructions. Six cytokines were measured once in duplicate by Cytometric Bead Array using a FACSCalibur flow cytometer (both BD, California, USA) in accordance with the manufacturer’s instructions.

### Statistical Analysis

Statistical analysis was performed using GraphPad Prism Software version 4 (Graphware Software Inc, San Diego, CA, USA). Categorical clinical characteristics of each cohort were compared using the Fisher’s exact test; continuous variable characteristics were compared using the Kruskall-Wallis test and are expressed as median and inter-quartile range [IQR]. Non-parametric data for *in vitro* studies are expressed as median (IQR) and compared using the Mann Whitney test. Trophoblast migration and clinical data were correlated using Spearman’s Rho (ρ). The AB assay on cohort 1, and concentrations of hormones and cytokines in pooled serum are expressed as mean and SD, and were analyzed using Mann-Whitney U for triplicate tests, or unpaired t-test for duplicate tests. In all cases P≤.05 denoted statistical significance. Intra- and inter-assay variability are expressed as mean and SD.

## Results

### Participant Characteristics at Enrolment

Cohort 1: Eleven women were included in each of the uninfected and *P. falciparum* infected groups. Parasitemia in infected women ranged from 0.02–0.2%. Every woman delivered a live neonate. Women with malaria infection had significantly lower median [IQR] hemoglobin levels than uninfected women (7.9 [7.3, 8.2] vs. 9.7 [8.0, 10.5] g/dL respectively, P = .02). Other variables including median maternal age (23 [19], [26] vs. 21 [20], [23] years, P = .9), weight (58 [50, 60] vs. 57 [53, 61] kg P = .8), gestational age at delivery (37 [35], [40] vs. 38 [36], [40] weeks, P = .6), cases of placental malaria (7/11 vs. 4/11, P = .4), birth weight (2.7 [2.4, 3.2] vs. 3.0 [2.6, 3.2] kg, P = .7) and use of chloroquine at enrolment (1/11 vs. 2/11 P = .6) were not different between infected and uninfected women, respectively.

Cohort 2: Thirteen participants with single *P. falciparum* infection and nine with *P. vivax* infection met the selection criteria, and were matched for gravidity and gestation to 13 uninfected women. Pregnancy outcomes were available for 33 (94%) of 35 participants. The mean gestational age at enrolment was 16±3 weeks and did not differ between groups, and 25 of 35 women (71%) were in their first pregnancy. Hemoglobin was significantly lower and parasitemia significantly higher in women with *P. falciparum* infection compared to uninfected women and those with *P. vixax* infection respectively (P<0.01; P = .0001; Table 1). Compared with uninfected women, women with *P. falciparum* infection at enrolment were more likely deliver a placenta with placental malaria (P = .01). Maternal age, weight at booking, current fever, use of anti-malarials in the last two weeks and birth outcome (gestational age, and birth weight) did not differ between the groups (Table 1).

**Table 1:** Clinical characteristics of cohort 2 at enrolment and delivery.

Clinical characteristic	Uninfected women(n = 13)	*P.v* infected women (n = 9)	*P.f* infected women(n = 13)	P value
Gest age (wks)	16 (12, 19)	18 (14, 20)	18 (13, 18)	NS
Maternal age (yrs)	21 (18, 24)	21 (19, 27)	21 (19, 27)	NS
Primigravidae	9/13	6/9	10/13	NS
Weight (kg)	52 (49, 55)	54 (51, 62)	55 (47, 59)	NS
Hb (g/dL)	10.2 (9.4, 11.2)	10.4 (9.1, 11)	8.9 (8.2, 9.7)**	P<.01
Parasitemia (parasites/ul)	–	128 (90, 160)	4160 (1120, 7700) ***	P = .0001
Malarial symptoms in last 2 days	1/13	1/9	2/13	NS
Taken anti-malarials in last 2 weeks	1/13	1/9	2/13	NS
Placental malaria at delivery	2/10	NA	7/9*	P = .02
Birth weight (kg)	2.7 (2.5, 3.2)	3.0 (2.7, 3.5)	2.7 (2.6, 2.8)	NS
Gest age	39 (38, 40)	40 (38, 42)	42 (39, 44)	NS

Note: all characteristics are expressed as media (IQR), or as frequency. The distribution of parasitemia was normalized by log transformation for subsequent analysis. Species diagnosis and parasitemia were determined by light microscopy, and placental malaria was defined as any malarial pigment found in fibrin deposits, infected red blood cells, or pigmented monocyte by histology on Giemsa stained placental sections. Anti-malarials use included sulphadoxine pyrimethamine, artemether, or chloroquine within the last two weeks. Malarial symptoms were reported within the last 2 days and included history of or current fever, pallor, chills and rigors, or body ache. Gestational age of cohort 2 was determined by ultrasound. Gestational age at delivery was estimated using the Ballard scoring method. Delivery outcomes were available for all participants, except one uninfected and one *P. vivax* infected woman. Placental histology was not available (NA) for any *P. vivax* enrolment cases, but was available for ten and nine deliveries that were uninfected and *P. falciparum* infected respectively at the time of enrolment.

### Pooled Serum from *P. falciparum*-infected Women Inhibits Trophoblast Invasion

**Figure 1:**
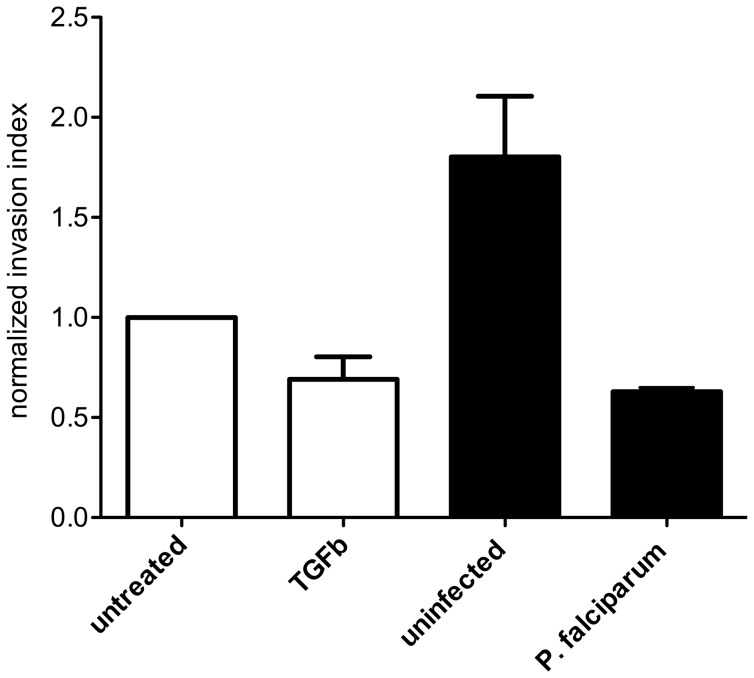
The effect of pooled serum from uninfected and *P. falciparum* infected women at enrolment on HTR8/SVneo invasion index over 48 h. Invasion index indicates the proportion of cells invaded through the Matrigel™, normalized to baseline invasion of trophoblast cells treated with complete media (untreated group). Serum from infected women inhibited median (IQR) invasion by 65% (Mann Whitney-U test P = .1) compared to serum from uninfected women, and by 37% compared with complete media treatment (P = .06). The malaria-specific reduction in invasion was comparable to that of 10 ng/ml TGFβ (positive control for invasion inhibition), which inhibited invasion by 31% (Mann Whitney U-test, P = .06). Normal uninfected pregnancy serum enhanced trophoblast invasion by 80% (P = .06) compared with complete media alone. Both viability and invasion assays were repeated three times in triplicate. Data shown are expressed as median and (IQR) of the mean of each of three independent experiments, repeated in triplicate.

Serum from *P. falciparum*-infected women inhibited HTR8/SVneo cell invasion by 37% compared to complete medium, and by 65% compared to treatment with serum from uninfected women, (P = .06 and P = .1 respectively, Figure 1). Compared with complete medium, TGFβ treatment (positive control) also reduced invasion by 31% (P = .06), validating the assay. There was a trend for uninfected pooled serum to promote invasion (80%, P = .06), compared to trophoblasts incubated with complete medium.

### Plasma from Individuals with *P. falciparum*, but not *P. vivax* Infection Inhibits Trophoblast Migration

**Figure 2:**
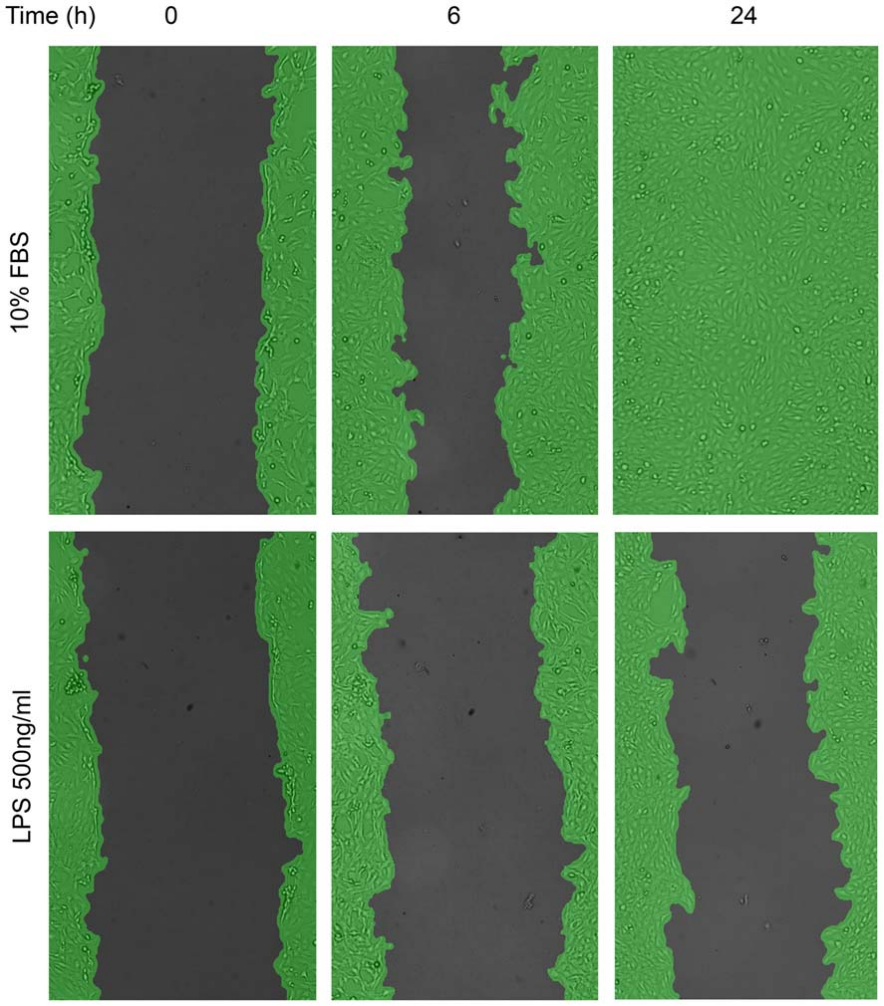
Migration of Swan cells was determined over time using Wimasis software. Swan 71 trophoblast cells were plated, and scratched 12 h later. At the time of treatment (0 h) with either 10% FBS, serum free media (SFM, not shown), or LPS 500 ng/ml in SFM, or with 10% plasma in SFM (not shown); three independent images were taken at 10x magnification. The same frames were imaged subsequently at 6 and 24 h following treatment. Images were up-loaded to www.ibidi.com, and the percent cell coverage (green) compared to the scratched area (black) was determined in each frame using WIMASIS analysis software. The percent increase in migration over time (Figure 3 A and B) was determined by subtracting cell coverage at 0 h from subsequent time points.

We tested the ability of plasma from women with or without malaria in early pregnancy to inhibit Swan 71 cell migration. By 24 h, cells treated with 10% FBS had recolonised the scratch area completely (Figure 2). LPS treatment reduced migration at 6 and 24 h by 73% and 70% respectively relative to controls in 10% FBS (P = 0.03 in both cases). Mean inter-assay variability in migration at 24 h was low (2±1.4%) as was mean intra-assay variability between replicates (3.1±1.7%).

**Figure 3:**
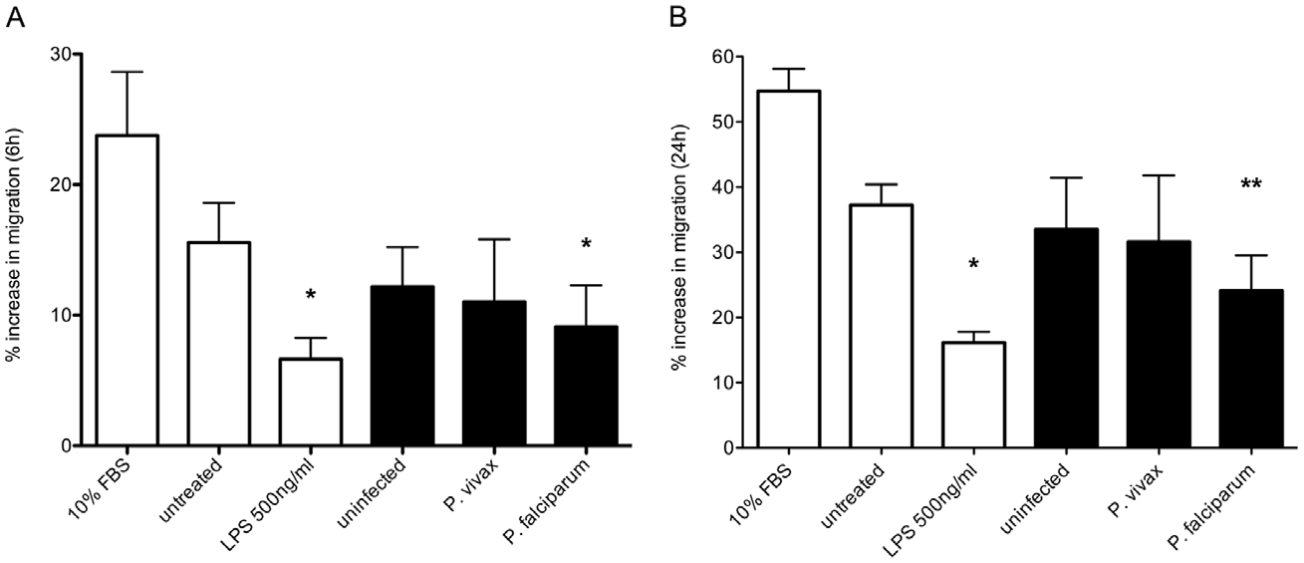
Plasma collected from *Plasmodium falciparum* infected women in early pregnancy inhibits Swan cell migration at 6 h (A) and 24 h (B). Median (IQR) percent increase in migration (relative to 0 h) of Swan 71 cells was measured over 24 h in response to control treatments (10% fetal bovine serum [FBS] in serum free medium, untreated [SFM], and 500 ng/ml LPS in SFM, open bars) or with 10% plasma from individuals (black bars) that were malaria-uninfected (n = 13), *P. vivax*-infected (n = 9) or *P. falciparum-*infected (n = 13), over 4 independent experiments. (A) At 6 h, plasma collected from women with *P. falciparum* infection inhibited migration compared with plasma from uninfected women (*P = .01). Treatment with LPS significantly inhibited migration (*P = .03) compared with 10% FBS treatment. There was no difference between uninfected and *P. vivax* plasma treatments (P = .35). (B) At 24 h, compared with cells treated with uninfected plasma, plasma collected from women with *P. falciparum* infection significantly reduced migration (**P = .004), while migration with plasma from *P. vivax* infection was unchanged (P = .64). LPS significantly inhibited migration compared to cells treated with 10% FBS (*P = .03).

Median migration of Swan 71 cells co-incubated with plasma from 13 women with *P. falciparum* infection was reduced by 20% at 6 h and 31% at 24 h compared with cells treated with plasma from 13 uninfected women (P = .01, P = .004, Figure 3 A and B respectively). Conversely, migration in cells treated with plasma from women with *P. vivax* infection (n = 9) did not differ significantly from uninfected pregnancy plasma at 6 and 24 h (P = .35 and P = .64, respectively).

### Pooled Serum or Plasma from *P.falciparum* Malaria-infected Women does not Reduce Trophoblast Viability

Blood serum or plasma from women with *P. falciparum* infection impaired migration and invasion of trophoblasts. We determined whether changes in trophoblast viability or metabolic status could account for impaired function using the AB viability assay.

**Figure 4:**
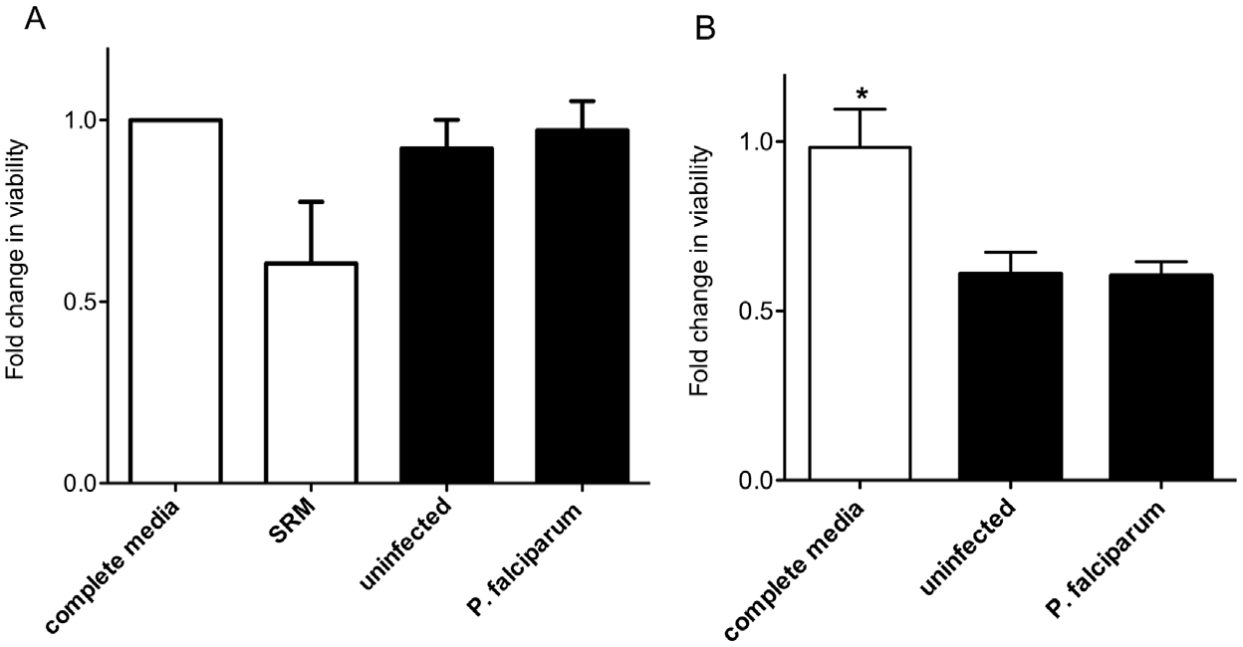
*Plasmodium falciparum* infection status does not affect trophoblast viability. (A) Mean and SD fold change in HTR8/SVneo viability relative to that of cells treated with complete media (open bars) was measured by percent reduction in AB dye after 4 h, following incubation with serum treatments (black bars) for 48 h. Compared with complete medium, complete medium supplemented with 10% volume pooled serum from women with or without infection had no effect on trophoblast viability (ANOVA P = .4). Treatment with SRM (open bar, positive control for a negative effect on trophoblast viability) reduced cell viability compared to trophoblasts treated with complete media (Mann Whitney test P = .06). Figure 4A indicates the mean and SD of each of three independent experiments, repeated in triplicate. (B) Median (IQR) fold change in viability of Swan 71 following treatment with plasma from individual women in early pregnancy. Swan 71 cells were treated once in triplicate for 24 h with either complete media (open bar), or serum free media supplemented with 10% plasma from women with (n = 13) and without (n = 11) *P. falciparum* infection in early pregnancy (black bars). Following treatment, Swan 71 cells were incubated with AB and cell metabolism (as a proxy for viability) was normalized to that of cells treated with complete media. There was no difference in viability between plasma treatments with infection (P = .4), but cells cultured in complete media had relatively higher measure of viability than those treated with 10% plasma (*P = .01 in both cases).

For the first cohort, the effect of infected serum on AB reduction by cells was determined by normalizing effects of SRM or pooled serum to complete medium, in three independent experiments. The mean (SD) fold change in activity after two days of treatment did not differ between cells treated with complete medium (1.0, control), or with serum from uninfected women (0.92 (0.08)) or malaria infected women (0.97 (0.08)) (ANOVA P = .4, Figure 4A). Treatment with SRM (positive control for reduced trophoblast mitochondrial activity because of restricted serum growth factors) significantly decreased AB reduction (0.61 (0.16); P = .05 compared to complete medium).

Compared to complete medium, cells treated with 10% plasma for 24 h had reduced activity in the AB assay (P = 0.01 for both uninfected [n = 11] and *P. falciparum*-infected [n = 13]), probably due to higher levels of metabolic stimulatory factors in FBS compared to adult plasma. Importantly, there was no difference in the ability of Swan 71 cells to reduce AB after treatment with uninfected or *P. falciparum* infected plasma (P = .4, Figure 4B).

### High Parasitemia Negatively Influences Migration

**Figure 5:**
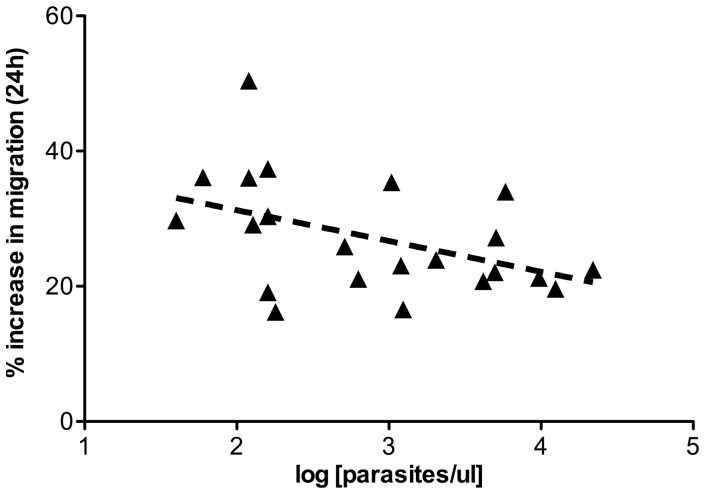
Parasite density negatively influences Swan 71 cell migration. Parasite densities from all *P. falciparum and P.vivax* infected participants (n = 22) were log transformed, and correlated with respective Swan 71 migration data at 24 h post-treatment. There was a significant negative association between density of infection in early pregnancy with migration (P = .02). No other clinical parameters were found to correlate with Swan 71 cell migration in response to plasma treatment.

To further explore the relationship between malaria infection and impaired trophoblast migration, we correlated clinical variables of individual participants with Swan 71 cell migration at 24 h. Among all participants, there was no apparent relationship between hemoglobin levels, gestational age at enrolment, or maternal characteristics (age, weight) with migration. However, among infected women, parasitemia with either species at time of infection was significantly inversely correlated with migration (n = 22, ρ = −.48, P = .023, Figure 5). There was a positive association between Swan 71 cell AB reducing power and migration (n = 24, ρ = .37, P = .08) but this did not reach significance.

### Association of Altered Maternal Levels of Invasion Regulatory Factors in Mothers with *P. falciparum* Infection in Early Pregnancy

To identify potential factors in *P. falciparum* malaria-infected pooled serum responsible for trophoblast invasion inhibition, we measured an array of cytokines and pregnancy-associated hormones known to modulate trophoblast invasion. Concentrations of invasion-promoting factors in serum from malaria-infected women including pregnancy-related hormones IGF-I, IGF-II and cytokine IL-8 [11] were lower than concentrations in serum from uninfected women [by 37% (P = .07), 25% (P = .1) and 47% respectively (P = . 09)] (Table 2).

**Table 2:** Concentration of invasion- modulating factors in pooled serum from uninfected and *P. falciparum* infected women at enrolment.

Analyte	reported effect on trophoblast invasion	mean (SD) conc. in uninfected women	mean (SD) conc. in *P.f* infected women	P value
IGF-I	+	180 (2)	114 (1)	0.07
IGF-II	+	1964 (82)	1470 (60)	0.1
IL-8	+	163 (3)	87 (15)	0.09
hCG	–	23 (1)	27 (1)	0.1
IL-10	–	5.2 (0.1)	17.4 (2.0)	0.08
IL-1β	–	not detected	2.2 (0.9)	–
TNFα	–	not detected	1.6 (0.4)	–
IL-6	+/−	4.2 (0.8)	4.4 (0.3)	–
IL-12p70	unreported	not detected	1.9 (0.9)	–

Note: Mean concentrations (and SD) of invasion stimulatory factors (IGF-I, IGF-II, and IL-8) were lower in pooled serum from women with *P. falciparum* infection compared to match uninfected controls. In the same pooled serum samples, factors that inhibit trophoblast invasion (hCG, IL-10) were higher in infected women than in uninfected women. IGF- Insulin like growth factor, IL- interleukin, TNF tumor necrosis factor, hCG human chorionic gonadotrophin. (+) indicates known previously reported trophoblast invasion-promoting effects [46], [47], [48], [49], [50], known trophoblast invasion inhibitory effects are indicated by (−) [48], [49], [51], [52], reports of both inhibitory and promoting effects are indicated by (+/−) [53], and (unreported) indicates an absence of previous reports on trophoblast invasion. All analyte concentrations in pooled serum were measured once and are expressed as pg/ml, except hCG (IU/ml). IGF-I, IGF-II and hCG were measured in triplicate (analyzed using the Mann-Whitney test), and all cytokines were measured in duplicate (analyzed using unpaired t test with Welch’s correction).

Conversely, concentrations of invasion-inhibitory factors including the hormone hCG and cytokine IL-10 were higher in serum from women with infection than in uninfected women (by 20%, P = .1 and by 340%, P = .08 respectively, Table 2). Concentrations of IL-6 were not different between the groups. Low levels (<3 pg/ml) of TNF, IL-1β and IL-12p70 were detected in infected serum but were undetectable in serum from uninfected women.

## Discussion

Because little is known about the pathogenesis of FGR associated with malaria in early pregnancy, we used an *in vitro* approach to indirectly investigate the impact of malaria infection on placental development.

Serum from *P. falciparum*-infected women in their early second trimester inhibited trophoblast invasion to an extent comparable to a known potent anti-invasion cytokine, TGFβ [11]. In a different but complementary *in vitro* model, factors in maternal blood from *P. falciparum-,* but not *P. vivax-*infected women also inhibited trophoblast migration. The differential effect of serum or plasma from uninfected and *P. falciparum-*infected pregnant women on trophoblast behavior could not be attributed to differences in trophoblast viability or maternal clinical characteristics (other than hemoglobin levels), suggesting the presence of factors that directly regulate EVT invasive and migration pathways in the blood of *P. falciparum -*infected women. This study is the first to provide evidence that infection with *P. falciparum* in the first half of pregnancy might interfere with placental development, and thereby contribute to FGR.

The inhibitory effect on trophoblast migration was most pronounced in women with high parasitemia, and was similar to that of exposure to LPS. This phenomenon could be due to a common inhibitory mechanism between pathogens, possibly through the activation of innate pattern recognition system [35], [36]. This hypothesis assumes direct interaction between migrating EVTs and parasite antigens within the maternal decidua. Alternatively, changes in maternal physiology (and therefore in the exudate that bathes EVTs migrating within the decidua) or immune activation in response to infection may have an indirect effect on EVT behavior.

Our data do not support an early pregnancy effect of *P. vivax* infection on trophoblast migration. This may be due to the inherently lower parasitemias observed with this species, or the presence of virulence factors specific to *P. falciparum* infection. However, the lack of association may be due to the limited sample size of P. vivax infections. Therefore further investigation in a larger population is required to fully exonerate early pregnancy *P. vivax* infection from having an impact on placental development.

In both cohorts, *P. falciparum*-infected women were more likely than uninfected women to be anemic. Previous studies associate moderate to severe anemia with increased trophoblast invasion [37]. This would suggest that anemia does not explain the decrease in invasion associated with serum from *P. falciparum* infected women in our study. Similar to previous reports [26], pregnancy serum from normal uninfected women promoted invasion, probably due to the high levels of invasion-stimulatory hormones and chemokines [11].

Inadequate trophoblast invasion may be common to both preeclampsia and malaria in pregnancy. However, in contrast to what we observed in our *in vitro* study of *P. falciparum* infected women, similarly powered studies of serum from pre-eclamptic women reported apoptosis in trophoblast cell lines [25], [30]. The molecular mechanisms underlying reduced trophoblast invasion with malaria requires further in depth study, and confirmation in vivo, but may be due to inappropriate maternal inflammatory responses, rather than apoptosis, as is observed with preeclampsia [38].

Blood flow to the intervillous spaces (and, therefore, the risk of placental malaria) commences late in first trimester, but the timing of potential effects of systemic *P.falciparum* infection on placental development may be different, extending potentially from conception to mid-pregnancy, as trophoblast invasion continues until this time [39]. Therefore, in addition to placental malaria, impaired placentation may contribute, by different pathophysiological mechanisms, to the development of FGR.

Our *in vitro* findings that *P. falciparum* infection impaired trophoblast invasion provide a mechanistic explanation to support recent observations that associate poor placental blood flow with reduced fetal growth [15]. Taken together these findings suggest a mechanism to explain how events in early pregnancy may underlie the subsequent development of hemodynamic disturbances, risk of hypertension [40] and placental insufficiency. These potential effects of malaria may act in concert with postulated mechanical processes (such as “clogging” of the intervillous spaces [18]) and pathophysiological changes in placental function induced by infection and inflammation of the intervillous space.

Elevated pro-inflammatory cytokines and reduced IGF hormones associated with malaria infection have been described at delivery [27], [29]. We extend those observations to show that early pregnancy the balance of hormones and pro-inflammatory cytokines circulating in malaria-infected women was associated with a stronger anti-trophoblast invasion and a weaker pro-invasion milieu. It is difficult to determine whether altered levels in early pregnancy predispose to, or are a consequence of, malaria infection. However, studies at term suggest these disturbances are a direct result of current infection and immune activation [41], rather than susceptibility, per se. It is likely that altered levels of some of these factors, or others, may explain the reduced trophoblast invasion observed *in vitro*. Overall, this finding suggests that *P. falciparum* malaria infection in early pregnancy can affect important determinants of placental development. Further studies with larger numbers of women with *P. vivax* malaria are needed to determine whether this infection also affects placental development.

This study was designed to determine if commonly used models of placental development were useful to test the potential impact of malaria in early pregnancy. The *in vitro* assays used are highly relevant to placental development, and have been the standard alternative to primary tissue for many studies [25], [33], [34], [42], [43] The results now suggest a need for studies to examine the migratory behavior of EVTs in vivo in infected women; if confirmed, further work would then be required to delineate the molecular interactions that underpin the effect of malaria infection on trophoblast. Explant cultures investigating the behavior of primary EVTs [44] would be of great interest. Although challenging, longitudinal examination of uterine artery blood flow by Doppler ultrasound and examination of term uterine bed biopsies at delivery to assess the timing and persistence of impaired trophoblast invasion *in vivo* would be useful to validate the *in vitro* findings.

The study of malaria in early pregnancy presents a unique set of clinical, ethical and experimental challenges. Because of the limited presentation of women to antenatal clinics in malaria-affected countries prior to second trimester [45], and the asymptomatic nature of infections in these areas, the true burden of malaria-attributable poor pregnancy outcomes due to early infection is difficult to determine, and remains an important, but inadequately addressed research area. Studies that seek to address the impact of malaria indirectly may be essential to developing better disease management strategies that adequately address protection from infection over the course of pregnancy.

### Conclusion

This study suggests a mechanistic link between *P. falciparum* malaria infection in early pregnancy and impaired placental development. This appears to be associated with higher parasitemia, and dysregulation of inflammatory and hormonal pathways that regulate extravillous trophoblast function during malaria infection, rather than decreased trophoblast viability. The potential for malaria infection in early pregnancy to cause placental insufficiency may be a further pathophysiological mechanism leading to malaria-associated FGR and needs urgent comprehensive evaluation to better inform the optimal timing of intervention strategies.
