# Correction: Does Malaria Affect Placental Development? Evidence from *In Vitro* Models

**DOI:** 10.1371/annotation/4faa7351-837e-4531-a513-0ea80277017f

**Published:** 2013-08-21

**Authors:** Alexandra J. Umbers, Danielle I. Stanisic, Maria Ome, Regina Wangnapi, Sarah Hanieh, Holger W. Unger, Leanne J. Robinson, Elvin Lufele, Francesca Baiwog, Peter M. Siba, Christopher L. King, James G. Beeson, Ivo Mueller, John D. Aplin, Jocelyn D. Glazier, Stephen J. Rogerson

A funding organization was incorrectly omitted from the Funding Statement. The first sentence of the Funding Statement should read: "This project was supported by the NHMRC (Project Grant 509185 to SJR and Project Grant 575534 to JB and SR)and by the Malaria in Pregnancy Consortium." 

